# Supplementary material for: Cellular taxonomy and spatial organization of the murine ventral posterior hypothalamus
Source: eLife. 2020 Oct 29;9:e58901. doi: 10.7554/eLife.58901 (PMC7595735; doi:10.7554/eLife.58901)
Supplement: Transparent reporting form [file elife-58901-transrepform.pdf]

1st Floor
24 Hills Road
Cambridge CB2 1JP, UK

P 01223 855340
W elifesciences.org
T @elife

eLife's transparent reporting form

We encourage authors to provide detailed information *within their submission* to facilitate the interpretation and replication of experiments. Authors can upload supporting documentation to indicate the use of appropriate reporting guidelines for health-related research (see EQUATOR Network), life science research (see the BioSharing Information Resource), or the ARRIVE guidelines for reporting work involving animal research. Where applicable, authors should refer to any relevant reporting standards documents in this form.

If you have any questions, please consult our Journal Policies and/or contact us: editorial@elifesciences.org.

Sample-size estimation

- You should state whether an appropriate sample size was computed when the study was being designed
- You should state the statistical method of sample size computation and any required assumptions
- If no explicit power analysis was used, you should describe how you decided what sample (replicate) size (number) to use

Please outline where this information can be found within the submission (e.g., sections or figure legends), or explain why this information doesn't apply to your submission:

-For scRNA-seq experiments, # animals and # single cells is described (Results, Materials and Methods and in figure legends), for all FISH, # animals and # cells and for and for viral tracing experiments, # animals is described (in Materials and Methods).
-Sample size/power analysis was not computed.

Replicates

- You should report how often each experiment was performed
- You should include a definition of biological versus technical replication
- The data obtained should be provided and sufficient information should be provided to indicate the number of independent biological and/or technical replicates
- If you encountered any outliers, you should describe how these were handled
- Criteria for exclusion/inclusion of data should be clearly stated
- High-throughput sequence data should be uploaded before submission, with a private link for reviewers provided (these are available from both GEO and ArrayExpress)

Please outline where this information can be found within the submission (e.g., sections or figure legends), or explain why this information doesn't apply to your submission:

eLife Sciences Publications, Ltd is a limited liability non-profit non-stock corporation incorporated in the State of Delaware, USA, with company number 5030732, and is registered in the UK with company number FC030576 and branch number BR015634 at the address 1st Floor, 24 Hills Road, Cambridge CB2 1JP | August 2014

eLIFE
1st Floor
24 Hills Road
Cambridge CB2 1JP, UK
P 01223 855340
W elifesciences.org
T @elife

-Replicates: For scRNA-seq experiments, 5 female and 5 male mice were processed in four independent pools. Analysis indicated high level of reproducibility and this is presented in the supplemental figures. For all other methods, # animals and # cells are provided (Results, Materials and Methods and figures legends).

-Data exclusions: For scRNA-seq experiments, poor quality cells were excluded from the analysis. These were cells with less than 2000 transcripts, fewer than 1000 genes, more than 50 hemoglobin transcripts and more than 15% mtRNA context. Genes with fewer than 3 counts in at least 3 cells were also excluded from downstream analysis. Further, putative doublets were removed from downstream analysis; supplementary analysis showing this process is available on GitHub. For viral tracing expts, mice were excluded from the behavioral analysis if bilateral injection sites were absent or off-target. All data exclusions described in text.

-Sequencing data: All the data is deposited into SRA and GEO and will be publicly available upon acceptance. The study accession number has been included in the manuscript Methods section.

Statistical reporting

- Statistical analysis methods should be described and justified
- Raw data should be presented in figures whenever informative to do so (typically when N per group is less than 10)
- For each experiment, you should identify the statistical tests used, exact values of N, definitions of center, methods of multiple test correction, and dispersion and precision measures (e.g., mean, median, SD, SEM, confidence intervals; and, for the major substantive results, a measure of effect size (e.g., Pearson's r, Cohen's d)
- Report exact p-values wherever possible alongside the summary statistics and 95% confidence intervals. These should be reported for all key questions and not only when the p-value is less than 0.05.

Please outline where this information can be found within the submission (e.g., sections or figure legends), or explain why this information doesn't apply to your submission:

-For scRNA-seq experiments, all statistical analyses are described in the Materials and Methods; the code is also available on GitHub. No formal hypotheses tests were conducted. Violin plots of many observations are used extensively throughout the manuscript to show the distribution of observations.

(For large datasets, or papers with a very large number of statistical tests, you may upload a single table file with tests, Ns, etc., with reference to sections in the manuscript.)

Group allocation

- Indicate how samples were allocated into experimental groups (in the case of clinical studies, please specify allocation to treatment method); if randomization was used, please also state if restricted randomization was applied
- Indicate if masking was used during group allocation, data collection and/or data analysis

Please outline where this information can be found within the submission (e.g., sections or figure legends), or explain why this information doesn't apply to your submission:

-For scRNA-seq experiments, FISH and viral tracing experiments, blinding was not used and samples were not randomized.

eLife Sciences Publications, Ltd is a limited liability non-profit non-stock corporation incorporated in the State of Delaware, USA, with company number 5030732, and is registered in the UK with company number FC030576 and branch number BR015634 at the address 1st Floor, 24 Hills Road, Cambridge CB2 1JP | August 2014

Additional data files ("source data")

- We encourage you to upload relevant additional data files, such as numerical data that are represented as a graph in a figure, or as a summary table
- Where provided, these should be in the most useful format, and they can be uploaded as "Source data" files linked to a main figure or table
- Include model definition files including the full list of parameters used
- Include code used for data analysis (e.g., R, MatLab)
- Avoid stating that data files are "available upon request"

Please indicate the figures or tables for which source data files have been provided:

-All of the commercial software used in the paper are listed in the methods and appropriately referenced. All the custom code for analysis will be made public on GitHub once the paper is accepted. The FASTQ files and raw expression matrices have been submitted to GEO and the embargo will be lifted when the paper is accepted.

-All of the commercial software used in the paper are listed in the Methods and appropriately referenced. All the custom code for analysis will be made public on GitHub once the paper is accepted.
